# The shape parameters of coal and gangue particles derived from 3D scanning

**DOI:** 10.1038/s41597-023-02019-z

**Published:** 2023-02-23

**Authors:** Daolong Yang, Jinjing Tang, Ningning Hu, Youtao Xia, Yanting Yu, Qianqian Huang

**Affiliations:** 1 School of Mechareonic Engineering, Jiangsu Normal University, Xuzhou, 221116 China; 2 Center for Tribology, Jiangsu Normal University, Xuzhou, 221116 China; 3 School of Mechareonic Engineering, Nanjing University of Science and Technology, Nanjing, 210094 China; 4 School of Mechareonic Engineering, Lanzhou University of Technology, Lanzhou, 730000 China

**Keywords:** Coal, Mechanical engineering

## Abstract

The irregular shape of mineral particles directly affects the angle of repose, bulk density and flow-properties, and the interaction behaviour between the particles and a contact surface. This paper presents a dataset of spatial data and shape parameters collected from 37 gangue particles and 135 anthracite coal particles, which come from the Shangzhuang Coal Mine. The particle surface models were obtained by a Wiiboox white light raster 3D scanner and Reeyee software. To obtain the scanning surface, each particle was scanned 8 times in different axial rotation directions. The final scanning model was obtained by stacking two scanning surfaces, and the shape parameters, such as length ratio, flatness ratio, and Zingg index, were obtained. This dataset is particularly useful for researchers and engineers who want to investigate the shape of coal and gangue particles or who want to test or benchmark measurement methods concerning the three-dimensional morphology of particles.

## Background & Summary

**Fig. 1:**
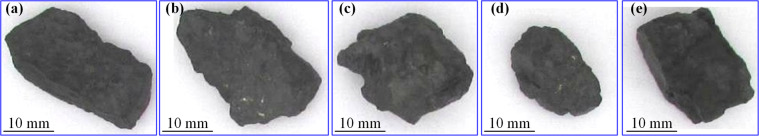
Five anthracite coal particles. (**a**–**e**) screening anthracite coal particles with particle size of 20–30 mm.

Shapes are of great interest for the physical and mechanical characterizations of coal particles. For instance, the bulk density^1^ and the angle of repose^2^, parameters that are widely reported in coal particle-related research, are greatly affected by the particle shape^3^. Figure 1a–e shows the different shapes of five anthracite coal particles. The shapes of anthracite coal particles are different, and there are few similarities. While the research community has proposed many methods for measuring particle shape^4–6^, the most commonly used is 3D scanning^7^. 3D scanning is widely used because it can adopt noncontact measurement, convenient reverse modelling, a large amount of data acquisition, and high contour accuracy.

The theoretical foundation of 3D scanning is to create a point cloud on the geometric surface^8,9^. These points can be used to interpolate the irregular surface shape. The denser the point cloud is, the more accurate the model (this process is called 3D reconstruction). The 3D scanning process is generally categorized as either contact 3D scanning or noncontact 3D scanning. Noncontact 3D scanning can be further categorized as grating 3D scanning (also known as photographic 3D scanning), X-ray CT scanning, and laser scanning; grating 3D scanning also includes white light scanning or blue light scanning, and laser scanning also includes point laser scanning, line laser scanning and surface laser scanning.

**Fig. 2:**
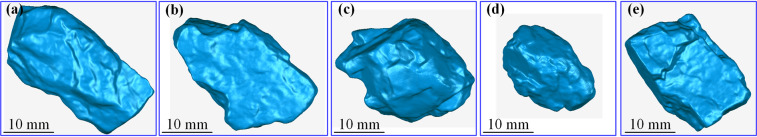
Five anthracite coal particle scanning models. (a-e) scanning model for screening anthracite coal particles with particle size of 20–30 mm.

**Fig. 3:**
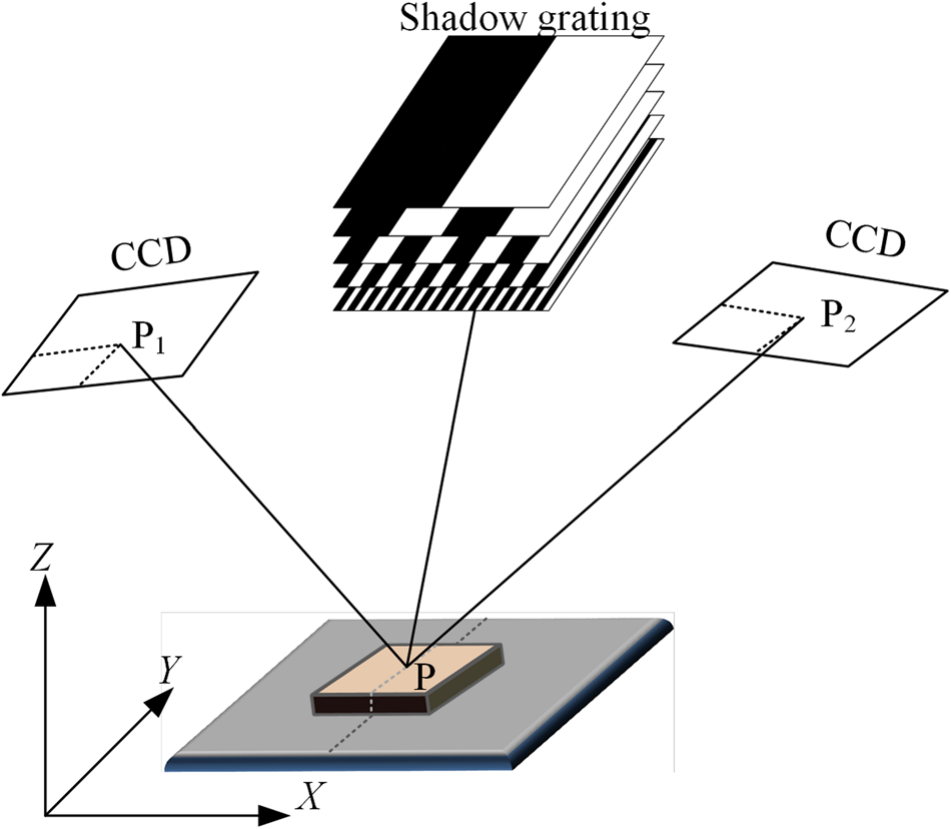
Working principle of white light grating 3D scanning.

Figure 2a–e shows the three-dimensional models of anthracite coal particles in Fig. 1a–e obtained by 3D scanning with white light grating, and its working principle is shown in Fig. 3^10^. The 3D scanning of white light gratings is mainly achieved with grating projection equipment and two industrial CCD cameras. The grating is projected on the object to be measured, and the thickness and displacement are changed. With the CCD camera, the captured digital image is processed through computer operation, and the actual 3D appearance of the object to be measured can be determined. The white light grating 3D scanner adopts noncontact white light technology to avoid contact with the object surface and can measure the models of various materials. During the measurement process, the measured object is placed on the platform, which rotates axially and allows the object to be measured from multiple perspectives. The system uses fully automatic splicing, which can easily realize high-precision measurements of the object. In addition, it can quickly obtain texture information and realistic object shapes while obtaining three-dimensional surface data, which can be quickly applied to scanning for manufacturing.

There are certain differences between the three-dimensional model and real particles when comparing Figs. 1a–e, 2a–e. These differences could be caused by light reflecting from the object surfaces, the interference of ambient light, or vibrations during object scanning. In addition, the model cannot completely scan the whole morphology of a particle in one particle scan. The particles are often spliced after multiple scans to form a three-dimensional model. The splicing process may also cause these differences. Additionally, the placement position of scanning particles is not necessarily the same every time and different scanning positions will result in different scanning surfaces.

To solve the above problems, researchers and engineers use a fixed scanning position, high-precision turntable and scanning calibration to reduce the differences between the three-dimensional model and real particles^11^. However, due to the heterogeneity of particle shapes, there is almost no available shape model database for coal particles. The respective research groups selected particles for 3D scanning for their own research, which lacks database support. To address the lack of a database for the shape models of coal particles, the data descriptor contains three-dimensional model scans of 135 anthracite coal particle models and 37 gangue particle models. It also describes the detailed steps of coal and gangue particles from actual particles to a three-dimensional model and proposes a method to improve the accuracy of the three-dimensional model.

## Methods

### Coal and gangue particles

**Fig. 4:**
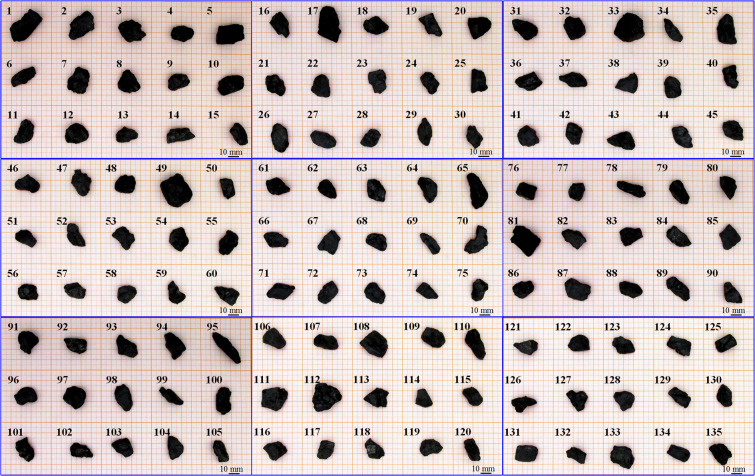
135 anthracite coal particles.

**Fig. 5:**
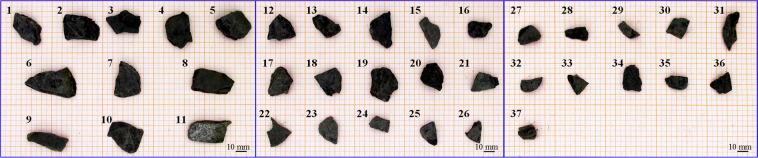
37 gangue particles.

A total of 135 anthracite coal particles and 37 gangue particles were investigated, which are screened, cleaned and dried, and shown in Fig. 4 and Fig. 5. Both anthracite coal and gangue particles come from the Shangzhuang Coal Mine, Gongyi City, Henan Province, China. The anthracite coal particles were randomly selected and the gangue particles were picked from the gangue mountain. The particle size ranges from 20–30 mm. Gangue particles are coal companions, a mixture of carbon, argillaceous and sandy shale, with low calorific value, and high hardness. They are solid waste in the mining industry^12^.

### Photography

High-pressure air is used to blow away any coal dust attached to the surface of the particles, and the particles are placed on coordinate paper and put into a camera box to take pictures, which are shown in datasets^13^. Using binarization processing and contour fitting, a two-dimensional contour of the particles can be obtained, which can be used for two-dimensional simulation^14,15^.

### Measurement process

**Fig. 6:**
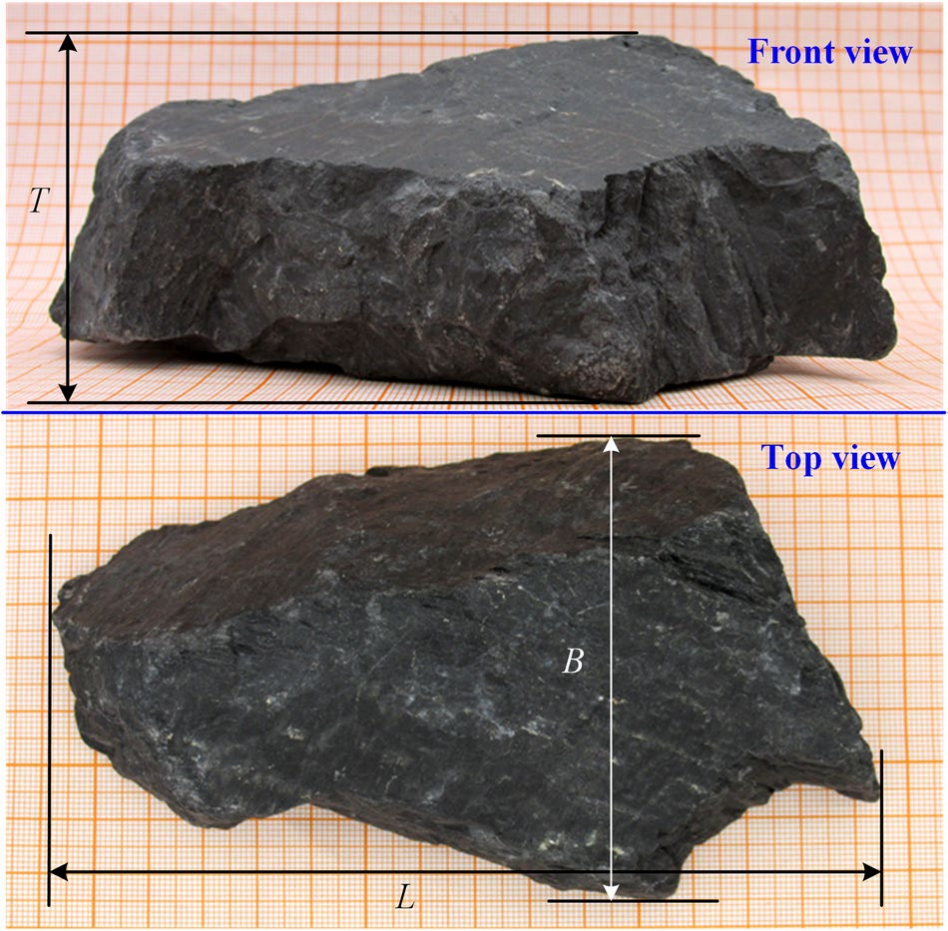
Triaxial diameter measurement method.

The particle triaxial diameter is usually regarded as an important fundamental parameter of particle shape^16^. The triaxial diameter measurement method of the particles is shown in Fig. 6. The particles are placed on a horizontal surface, and Vernier callipers are used to measure the length *L*, width *B*, and thickness *T* of the particles in the triaxial direction orthogonal to each other in a steady state. Specially, the length *L* is the maximum distance of the particle in the front view; the width *B* is the maximum distance of the particle perpendicular to the length *L*; and the thickness *T* is the distance between the upper and lower held particle^1^.

Usually, to further characterize the morphology of the particles, indicators such as uniformity, filling degree, and sphericity are used.

### Uniformity

The uniformity represents the ratio of two particle dimensions and consists of the length ratio *N*_*L-B*_ and flatness ratio *N*_*B-T*_, which are defined as shown in Eq. (1). The ratio *F* of length *N*_*L-B*_ to flatness *N*_*B-T*_ is called the Zingg index^17,18^, as shown in Eq. (2).

1 $$\left\{\begin{array}{l}{N}_{L-B}=L/B\\ {N}_{B-T}=B/T\end{array}\right.$$

2 $$F={N}_{L-B}/{N}_{B-T}=LT/{B}^{2}$$

When Zingg index *F* > 1, the particle shape should belong to the stick shape. The larger Zingg index is, the more elongated the particle shape is. When Zingg index *F* < 1, the particle shape should be flaky. The smaller Zingg index is, the more serious the flakiness of the particle is.

#### Filling degree

The filling degree is divided into the volume filling degree and the area filling degree^19^. The volume filling degree *F*_*V*_ is defined as the ratio of the volume of the outer rectangle of the particle to the actual volume of the particle, as shown in Eq. (3); the area filling degree *F*_*A*_ is defined as the ratio of the area of the outer rectangle of the vertical projection of the particle to the actual projection area of the particle, as shown in Eq. (4).

3 $${F}_{V}=LBT/{V}_{p}$$

4 $${F}_{A}=LB/A$$

The actual volume of the particles can be approximated by the volume of the particle model after 3D scanning, and the actual projected area of the particles can be approximated by the area of the coordinate paper occupied by the particle projection using the photographic pictures of the particles.

#### Sphericity

The sphericity indicates how close the particles are to a sphere and is also a widely used particle shape parameter, which describes the shape difference between spherical and nonspherical particles. There are many formulas used to define the sphericity^20,21^. Since the volume and surface area of the particle model are easily obtained in postprocessing of the 3D scanning model, the sphericity degree is shown in Eq. (5).

5 $${\varPsi }_{S}=\pi {D}_{V}^{2}/S$$

where

6 $${D}_{V}=\sqrt[3]{\frac{6{V}_{p}}{\pi }}$$

where *ψ*_*S*_ is the sphericity degree of the particle; *D*_*V*_ is the equivalent volume diameter of the particle; *V*_*P*_ is the volume of the particle; and *S* is the surface area of the particle.

### Experimental procedure

**Fig. 7:**
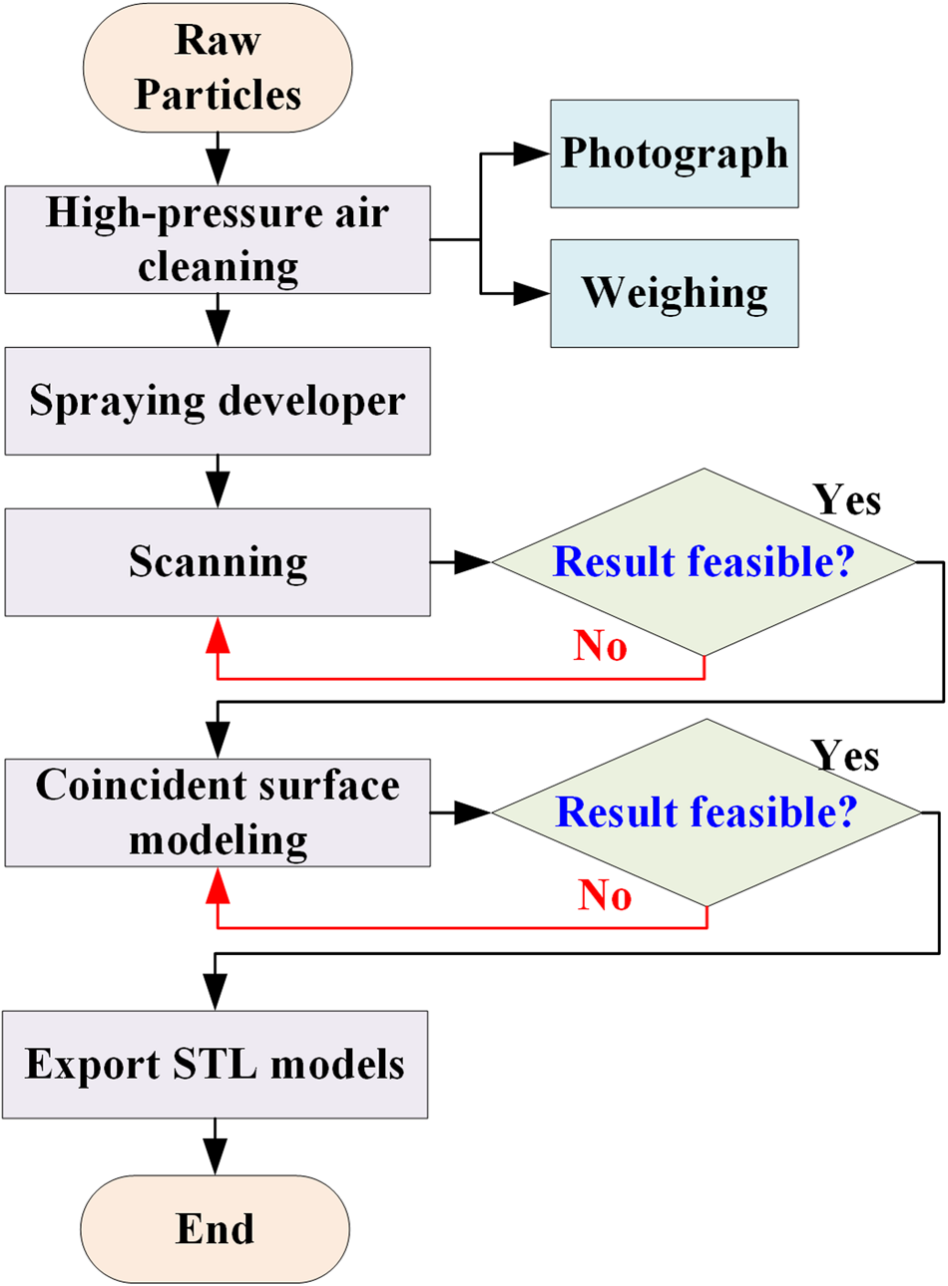
Scanning process.

**Fig. 8:**
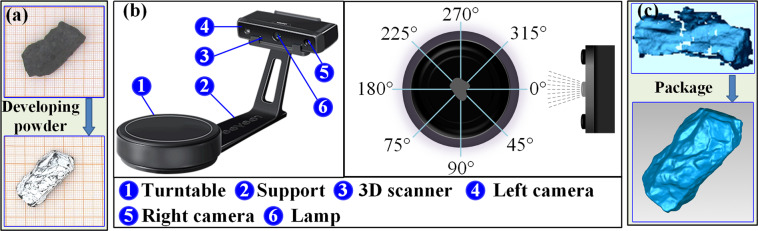
Experimental setup. (**a**) particle development, (**b**) experimental device, (**c**) scanning package.

The Wiiboox white light raster 3D scanner and Reeyee_v2.5.0 software were used to scan the particles and obtain the surface model of the particles. The scanning process is shown in Fig. 7, and the particles went through several steps, such as cleaning, spraying, scanning, splicing and exporting to the model. As the surface of coal particles is rough and dark, the reflection does not return when scanning, resulting in poor quality of the scanned data. Therefore, the coal particles also need a surface spraying developer, as shown in Fig. 8a. The developer is dye penetrant inspection materials. Before scanning the particles, shake the developer well, spray 150–300 mm from the particles, ensure that the particle surface is evenly sprayed with developer, and then wait the developer was dry on the surface of the particles. The Wiiboox white light raster 3D scanner is shown in Fig. 8b, including a turntable, fixed bracket and 3D scanner. The 3D scanner consists of two sets of CCD cameras and raster lights. The 3D scanner is installed with a fixed bracket between the scanner and the turntable, which ensures a stable distance between the CCD cameras and the measured particles. The turntable was automatically rotated 45° for each measurement, each measurement lasts 20–30 s, each particle was scanned 8 times in different directions, and the scanned model was obtained by superimposing the 8 scanned surfaces. The obtained scanning model was initially compared with the particle to determine whether the scanning and stitching were successful. Then, the particles were reversed, and the bottom and top of the particles, which were not scanned, were scanned again 8 times in different directions. The second scanning model was obtained by superimposing the 8 scanned surfaces, and the two scanning models were automatically stitched together to obtain the final model and judge the success of the model again, as shown in Fig. 8c. Finally, the acceptable particle scanning model was exported in STL format.

After exporting the particle models in STL format, Geomagic Studio 12 software was used to analyse and correct the particle models, addressing nonflow edges, self-intersections, highly refractive edges, spikes, small groups, small channels, and small holes. Geomagic Studio 12 software can obtain the number of units, volume, and surface area of the corrected particle model^13^.

## Data Records

The datasets were made available from Figshare 10.6084/m9.figshare.20231085.v4^13^. There are six zip files and one “Read me” text file in datasets, among which, Data.zip are the coal and gangue particles measurement and scanning data files, Photos.zip are the coal and gangue particles photo original files and processed files, coal-stl.zip and gangue-stl.zip are the coal and gangue particles scanning model original files in STL format, coal-wrp.zip and gangue-wrp.zip are coal and gangue particle scanning model processed files.

## Technical Validation

### Experimental error analysis

The coal and gangue particles in the experiment were randomly selected, and the error of the scanning results should come from the scanning method, splicing algorithm, etc. To obtain a more accurate particle model, scholars and engineers can use other scanning methods and more accurate splicing algorithms based on the scanning process in Fig. 7 of this paper to reduce the experimental error.

### Reliability of scanning results

Before scanning, the particles under test are photographed on standardized coordinate paper, and this can provide data on particle projection, including particle projection area, particle length and width dimensions, which can be compared with the scanning models. According to the scanning models using the required accuracy, if the error between the scanning models and the actual particles does not meet the required accuracy for use, the particle scanning can be reperformed. The error used for this dataset was 5%, and when the projected area length and width scale of the scanned model was within ± 5% of the error of the photographed particles, it was considered an acceptable result.

## Usage Notes

It can be helpful to convert STL meshes from binary to ASCII encoding, as some software does not support binary STL files.

## Data Availability

The device and software used to generate the 3D scanning models are Wiiboox white light raster 3D scanner and Reeyee_v2.5.0. The software used to generate the parameters of 3D scanning models are Geomagic Studio 12 and Microsoft Excel 2016.
